# GERIATRIC MEASURES OF PHYSICAL FUNCTION HAVE THEIR ROOTS IN EARLY CHILDHOOD BRAIN HEALTH

**DOI:** 10.1093/geroni/igad104.2795

**Published:** 2023-12-21

**Authors:** J Kathy Xie, Lauren Winslow, Avshalom Caspi, Terrie Moffitt

**Affiliations:** Duke University, Durham, North Carolina, United States; Duke University, Durham, North Carolina, United States; Duke University, Durham, North Carolina, United States; Duke University, Durham, North Carolina, United States

## Abstract

Midlife is a pivotal period in the life course in terms of investing resources to mitigate challenges that can come in older age. It is important to understand the relationship between childhood and midlife health, as it could have predictive properties for aging. We investigated the association between early childhood brain health and midlife physical function in the Dunedin Study, a longitudinal birth cohort (n=1,037). Brain health was measured when Study members were age 3 using a neurological examination, tests of cognitive functioning, and temperament rating. Physical function was measured at age 45 using gait speed, step-in-place, chair stands, balance, and grip strength tests. Children with worse brain health had significantly worse physical function as adults (β, 0.23; 95% CI, 0.16 to 0.30; P < .001), after controlling for sex and childhood socioeconomic status. This relationship supports a link between cognitive and physical function in the aging and development process, the reconceptualization of aging and development on the same ontological path, and the utility of physical function tests – which can be used in many clinical settings – as reflections of lifelong, integrated health in addition to indicators of musculoskeletal integrity in older adults.

